# Non-proteolytic activity of 19S proteasome subunit RPT-6 regulates GATA transcription during response to infection

**DOI:** 10.1371/journal.pgen.1007693

**Published:** 2018-09-28

**Authors:** Abiola O. Olaitan, Alejandro Aballay

**Affiliations:** Department of Molecular Microbiology & Immunology, Oregon Health & Science University, Portland, United States of America; The University of Texas Health Science Center at Houston, UNITED STATES

## Abstract

GATA transcription factors play a crucial role in the regulation of immune functions across metazoans. In *Caenorhabditis elegans*, the GATA transcription factor ELT-2 is involved in the control of not only infections but also recovery after an infection. We identified RPT-6, part of the 19S proteasome subunit, as an ELT-2 binding partner that is required for the proper expression of genes required for both immunity against bacterial infections and recovery after infection. We found that the intact ATPase domain of RPT-6 is required for the interaction and that inhibition of *rpt-6* affected the expression of ELT-2-controlled genes, preventing the appropriate immune response against *Pseudomonas aeruginosa* and recovery from infection by the pathogen. Further studies indicated that SKN-1, which is an Nrf transcription factor involved in the response to oxidative stress and infection, is activated by inhibition of *rpt-6*. Our results indicate that RPT-6 interacts with ELT-2 *in vivo* to control the expression of immune genes in a manner that is likely independent of the proteolytic activity of the proteasome.

## Author summary

The conserved GATA transcription factor ELT-2 plays an important role in the control of genes required for both defense and recovery from infection. We show that RPT-6, a component of the 19S subunit, physically interacts with ELT-2 *in vivo*, controlling the expression of ELT-2-dependent genes and the response of the nematode *Caenorhabditis elegans* to bacterial infection. The proteolytic activity of the proteasome has surfaced as a key regulator of gene expression, but our results provide evidence indicating that a non-canonical activity of the 26S proteasome subunit plays an important role in the control of gene expression during the response to bacterial infection.

## Introduction

The regulation of gene transcription plays crucial roles in the control of an array of critical biological processes, including immune responses against microbial infections [1,2]. At the heart of immune activation are transcription factors, which directly bind to specific DNA motifs in promoter regions to control gene transcription. The GATA transcription factor ELT-2 is a major component of innate immunity and lies downstream of the conserved PMK-1/p38 mitogen-activated protein kinase (MAPK) signaling pathway in the nematode *Caenorhabditis elegans* [3]. Indeed, ELT-2 has been demonstrated to control immune responses against several human bacterial pathogens, including *Pseudomonas aeruginosa*, *Salmonella enterica*, *Enterococcus faecalis*, and *Cryptococcus neoformans*. [4–7]. Furthermore, ELT-2 has recently been shown to be involved in the control of host changes that also take place during recovery from bacterial infections [6,7]. Despite the several aforementioned studies describing the importance of ELT-2 in the regulation of myriad target genes required for response and recovery from infections, nothing is known about the mechanisms involved in the control of ELT-2 transcriptional activity.

The 26S proteasome has surfaced as a key regulator of gene expression, mostly via its proteolytic activity. However, increasing evidence suggests a non-proteolytic role of the 26S proteasome or its sub-complex, notably the 19S regulatory subunit, in the control of gene transcription [8,9]. This non-canonical activity of the 26S proteasome has been linked to the control of various aspects of gene transcription, including initiation and elongation steps, and chromatin remodeling [10–12]. Recent biochemical and genetic studies have shown that the proteasome can physically interact with transcription factors and regulate their interactions with coactivators as well as promoter regions, all leading to the control of gene activation [8,10,13]. For example, in yeast and mammalian cells, SUG1/RPT6 and some other proteasome subunits can physically interact with transcription factors to control gene transcription in a non-proteolytically fashion [8,14–17]. Also, viral gene transcription has been associated with the non-proteolytic activity of the 19S subunits during gene expression [11,18], but the role of non-canonical functions of the proteasome in the control of defense against pathogens and activation of innate immunity has not been studied.

We identified RPT-6, a component of the 19S proteasome subunit, as a binding partner of ELT-2. We showed that inhibition of *rpt-6* leads to inactivation of ELT-2-regulated immune genes during *P*. *aeruginosa* infection, resulting in enhanced susceptibility to the pathogen, similar to that observed in animals deficient in *elt-2*. We also demonstrated that both *elt-2* and *rpt-6* work together to control recovery after *P*. *aeruginosa* infection has been cleared. Disruption of the proteasome complex, but not the inhibition of proteasomal activity, prevents ELT-2 transcriptional activation of immune genes. Finally, we demonstrate that both RPT-6 and ELT-2 physically interact *in vivo* in *C*. *elegans* and that this interaction is affected by other components of the proteasome complex. Our findings show that the proteasome interacts with the transcription factor ELT-2 to control the activation of immune genes, extending our knowledge of the role of the proteasome in gene transcription to innate immunity.

## Results

### Identification of candidate ELT-2-interacting partners

As a first step to further understand the mechanisms by which ELT-2 controls gene expression, we attempted to identify potential interacting proteins with ELT-2/GATA during infection of *C*. *elegans*. A *C*. *elegans* strain carrying a stably integrated ELT-2::GFP transgene was exposed to *P*. *aeruginosa* prior immunoprecipitation of GFP-tagged ELT-2. A total of 14 candidate ELT-2-interacting proteins were identified using liquid chromatography-tandem mass spectrometry (LC–MS/MS) (S1 Table)

To validate the role of the potential ELT-2 partners in the control of innate immunity, we studied the susceptibility to *P*. *aeruginosa* of animals in which 10 candidate genes were inhibited by RNAi using all the commercially available clones to inhibit genes in *C*. *elegans*. Because ELT-2 is essential for *C*. *elegans* larval development [19], we reasoned that genes encoding the candidate binding partner would also be essential. Thus, we performed RNAi to downregulate *elt-2* and candidate genes at late larval stage 4 (L4). RNAi against four of the genes led to susceptibility to PA14 infection in the animals. However, only *rpt-6(RNAi)* displayed a very robust enhanced pathogen susceptibility that was comparable to that exhibited by *elt-2*(*RNAi*) animals (Table 1, S1A Fig). As ELT-2 transcriptional activity has been linked to recovery from acute bacterial infection [6,7], we reasoned that potential ELT-2 partners may also affect recovery from bacterial infection. We studied the survival of animals in which the candidate genes were knocked down by RNAi, infected with *P*. *aeruginosa*, and treated with streptomycin. We found that *rpt-6(RNAi)* animals failed to recover after *P*. *aeruginosa* infection (Table 1, S1B Fig). RNAi downregulation of *gpb-1* also enhanced susceptibility to infection and affected recovery. Inhibition of *gpb-1* causes defects in the body wall muscles [20], which may prevent bacterial clearance and affect recovery. Thus, this gene was not further analyzed and we focused on *rpt-6*, which like *elt-2*, controls both response to infection and recovery.

**Table 1:** *P*. *aeruginosa* infection and recovery assays following RNAi of genes for proteins that interact with ELT-2 during infection.

Gene	Median survival in PA14 (hours)	P-value	Phenotype	Median survival after PA14 recovery (days)	P-value	Phenotype
EV	52±6.93	-	WT	>3	-	WT
*elt-2*	28±6.93	P<0.0001	ESP	1.7±0.58	P<0.0001	ESR
*rpt-6*	36±0	P<0.0001	ESP	2.3±0.58	P<0.0001	ESR
*W10C8*.*5*	52±6.93	0.8344	WT	>3	0.1303	WT
*rab-8*	56±6.93	0.6761	WT	>3	0.5738	WT
*eif-3*.*E*	80±6.93	P<0.0001	ERP	>3	0.7172	WT
*tba-4*	44±6.93	0.0052	ESP	>3	0.4051	WT
*gpb-1*	40±6.93	0.0014	ESP	2±0.00	P<0.0001	ESR
*unc-52*	48±0	0.07	WT	>3	0.9689	WT
*F48E8*.*3*	52±6.93	0.1979	WT	>3	0.4516	WT
*hsp-12*.*2*	60±0	0.5259	WT	>3	0.0914	WT
*K02D7*.*1*	44±6.93	0.0462	ESP	>3	0.1421	WT

Empty vector (EV) and *elt-2* RNAi were used as controls, and *fer-1(b232ts)* animals were used in the assays. WT: wild type; ESP: enhanced susceptibility to *P*. *aeruginosa*; ERP: enhanced resistance to *P*. *aeruginosa*; ESR: enhanced susceptibility to *P*. *aeruginosa* recovery.

### Knockdown of *rpt-6* leads to loss of immunity through inactivation of ELT-2 transcriptional activity

**Fig 1:**
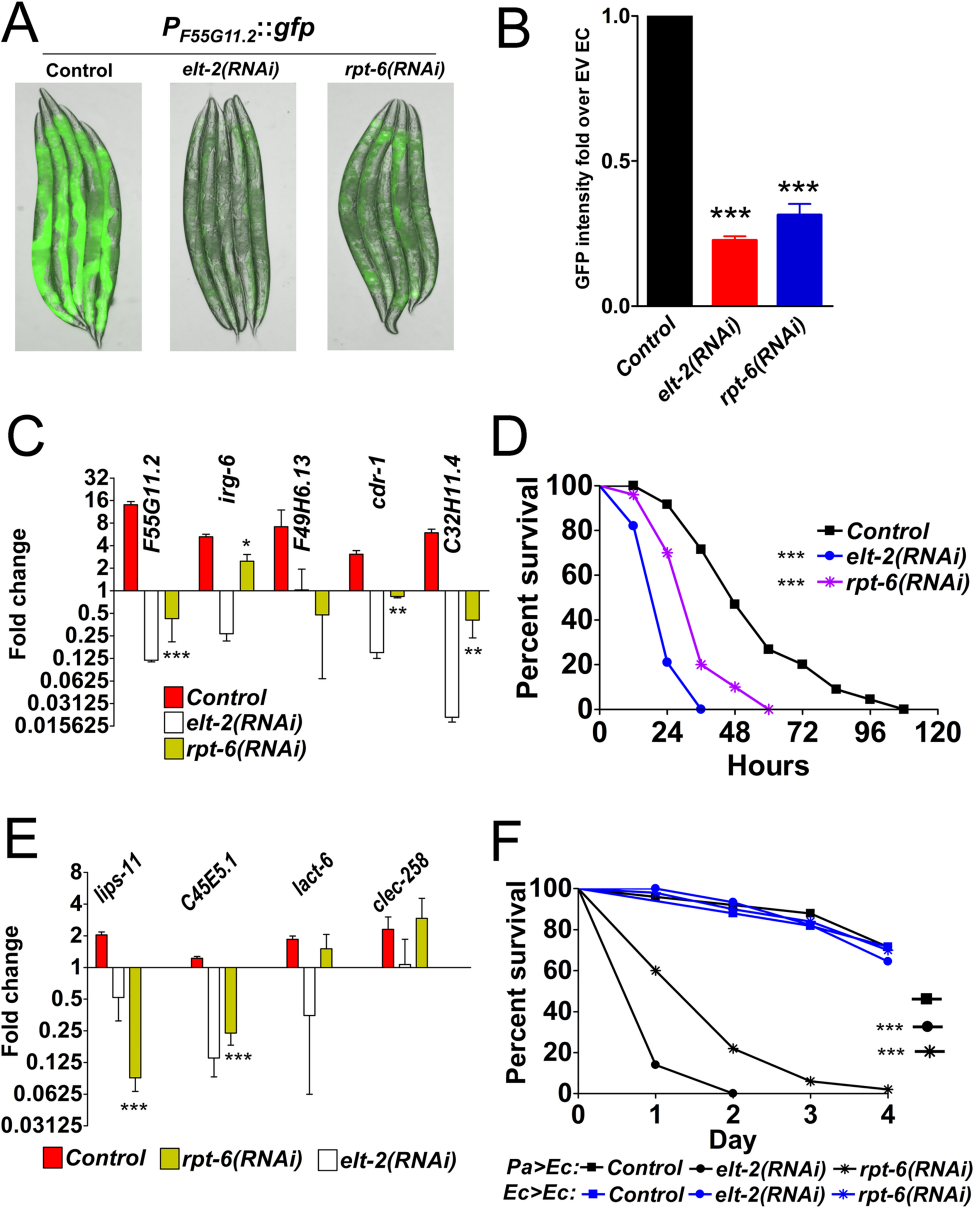
RPT-6 is required for activation of immune genes during infection. **A.** Control or RNAi treated *P*_*F55G11*.*2*_::*gfp* animals were grown on *E*. *coli* OP50 for 12 hours. **B.** Fluorescence images were quantified using ImageJ software. Bars represent means ± SEM; n = 3 (t-test, ***P<0.0001). **C.** qRT-PCR analysis of immune genes in *elt-2(RNAi)* or *rpt-6(RNAi)* animals exposed to *P*. *aeruginosa* for 12 hours relative to control animals exposed to *E*. *coli*. Red bars correspond to gene expression in control animals exposed to *P*. *aeruginosa* for 12 hours relative to control animals exposed to *E*. *coli*. Error bars indicate means ± SEM; n = 3 (t-test *P<0.05, **P<0.01, ***P<0.001). **D.** Control, *elt-2(RNAi)*, and *rpt-6(RNAi)* animals were exposed to *P*. *aeruginosa* and scored for survival, ***P<0.0001. **E.** qRT-PCR analysis of recovery genes *elt-2(RNAi)* or *rpt-6(RNAi)* animals exposed to *P*. *aeruginosa* and then recovered on *E*. *coli* plus streptomycin, relative to control animals on *E*. *coli* followed by *E*. *coli* plus streptomycin. Red bars correspond to gene expression in control animals exposed to *P*. *aeruginosa* and then recovered on *E*. *coli* plus streptomycin, relative to control animals on *E*. *coli* followed by *E*. *coli* plus streptomycin. Error bars indicate means ± SEM; n = 3 (t-test ***P<0.001). **F.** Control, *elt-2(RNAi)*, and *rpt-6(RNAi)* animals were exposed to *E*. *coli* (Ec) or *P*. *aeruginosa* (Pa) for 12 hours, treated with streptomycin, and then transferred to *E*. *coli* plus Streptomycin plates and scored for survival. Scoring started 24 hours post initial exposure to *E*. *coli* or *P*. *aeruginosa*, ***P<0.0001.

To study the mechanism by which RPT-6 controls immune activation, we first used a *P*_*F55G11*.*2*_::*gfp* transcriptional reporter strain that expresses GFP under the control of the promoter of *F55G11*.*2*, which is an ELT-2-dependent gene [3,4]. Inactivation of *elt-2* by RNAi usually leads to downregulation of the basal expression of *F55G11*.*2* [3]. Upon *rpt-6* RNAi, we observed a reduction of GFP expression in *P*_*F55G11*.*2*_::*gfp* animals similar to that observed when *elt-2* is also inhibited (Fig 1A; Fig 1B). Next, we used qRT-PCR to examine the effect of *rpt-6* inactivation on the induction of selected immune genes that contain the TGATAA ELT-2 binding motif in their proximal promoter regions and that are usually activated during *P*. *aeruginosa* infection [7]. Four out of the five examined immune genes failed to be activated during *P*. *aeruginosa* infection in both *elt-2(RNAi)* animals and *rpt-6(RNAi)* animals (Fig 1C). Figs 1D and S1A show that although *rpt-6(RNAi)* animals were not as susceptible as *elt-2(RNAi)* animals to *P*. *aeruginosa* infection, they were significantly more susceptible than the control animals (P<0.0001). The overall expression of immune genes in *elt-2(RNAi)* animals is not different from that of *elt-2(RNAi);rpt-6(RNAi)* animals (S2 Fig). S3A and S3B Fig shows that *elt-2* and *rpt-6* were effectively inhibited in the *elt-2(RNAi)* and *elt-2(RNAi);rpt-6(RNAi)* animals. Next, we aimed to determine whether *rpt-6* also played a role during recovery from bacterial infection. While RNAi downregulation of *rpt-6* prevented the activation of two out of the four tested genes involved in recovery from *P*. *aeruginosa* infection (Fig 1E), *rpt-6(RNAi)* animals failed to recover from the infection (Fig 1F). These results suggest that *rpt-6* and *elt-2* may work together to control the expression of a subset of immune genes.

Knockdown of *elt-2* or *rpt-6* leads to larval arrest [21]. To study whether the enhanced pathogen susceptibility of *rpt-6(RNAi)* animals is a consequence of the animals being sickly, we performed lifespan assays on *rpt-6(RNAi)* and *elt-2*(*RNAi*) animals. The result shows that both *elt-2(RNAi)* and *rpt-6(RNAi)* animals displayed comparable lifespans, although a small reduction was observed compared to control animals (S4 Fig). However, the rate of death of *elt-2(RNAi)* and *rpt-6(RNAi)* animals exposed to *P*. *aeruginosa* is higher than that of animals exposed to killed *E*. *coli* (Fig 1D and S4 Fig). These results indicate that the enhanced pathogen susceptibility is not simply a consequence of the animals being sickly, and are in agreement with other studies indicating that *elt-2* also controls lifespan in *C*. *elegans* [5,22,23].

Finally, we were interested in determining whether other components of the 26S proteasome also affected ELT-2 transcriptional activity. Thus, we examined the fluorescence expression of *P*_*F55G11*.*2*_::*gfp* animals after knocking down the proteasome genes *rpt-3*, *pbs-2*, *pas-6*, and *rpn-11*. RNAi of these genes, which belong to the three 26S sub-complexes (19S lid, 19S base, and 20S core), reduced *P*_*F55G11*.*2*_::*gfp* fluorescence (S5A Fig). We also examined whether another component of the 26S proteasome, RPN-11, was involved in the control of expression of ELT-2-dependent genes. As shown in S5B Fig, *rpn-11(RNAi)* animals failed to activate ELT-2 regulated-immune genes during *P*. *aeruginosa* infection, with the exception of *irg-6*. Consistent with these results, *rpn-11(RNAi)* animals were also susceptible to *P*. *aeruginosa* infection (S5C Fig). Thus, we concluded that *rpt-6* and other components of the 26S mediate ELT-2 transcriptional activity against *P*. *aeruginosa* infection.

### SKN-1 partially enhances immunity in *rpt-6*-inactivated animals

**Fig 2:**
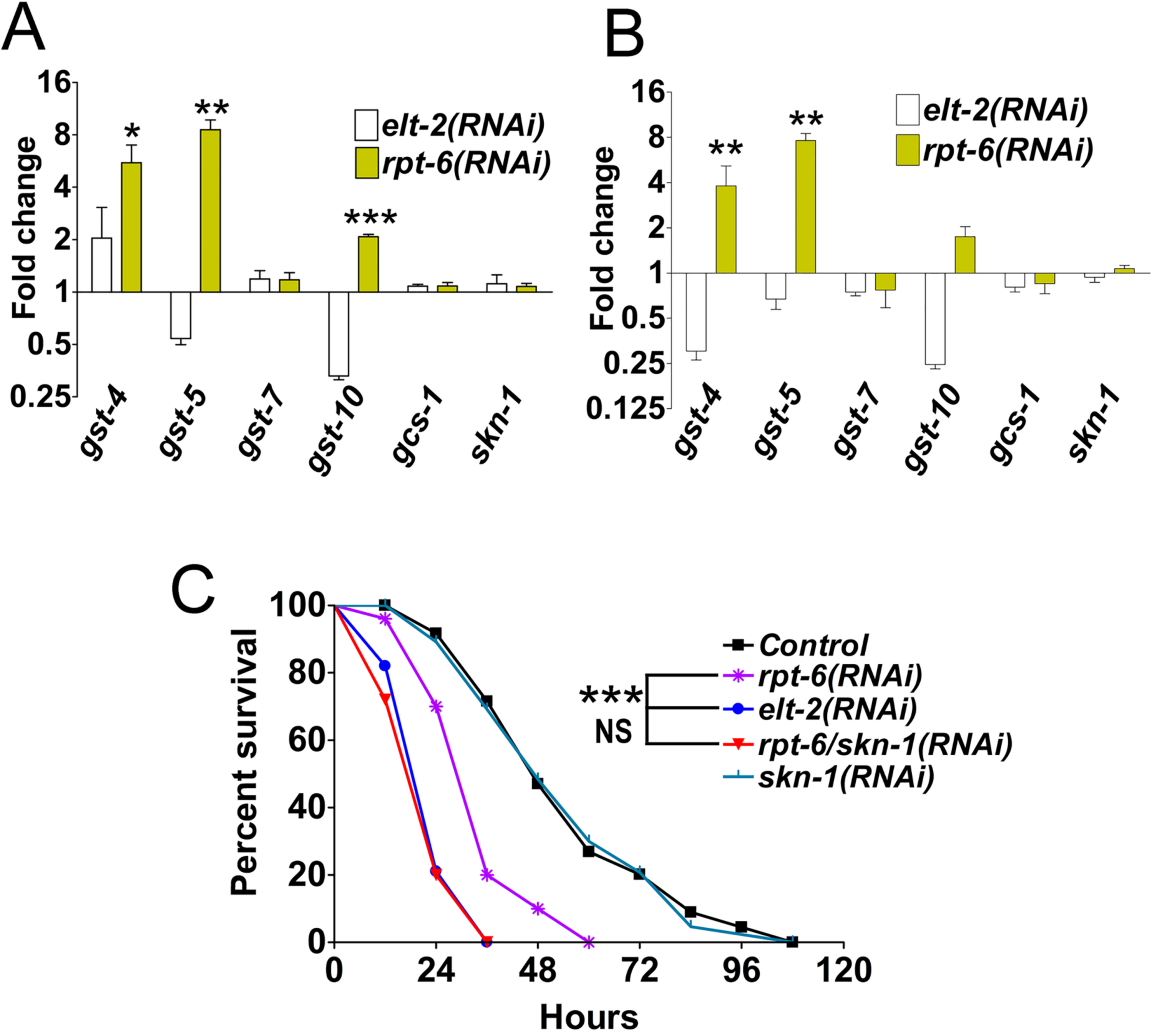
Knockdown of *rpt-6* activates a subset of SKN-1-dependent target genes and enhances survival against infection. **A.** qRT-PCR analysis of SKN-1-dependent genes in *rpt-6(RNAi)* or *elt-2(RNAi)* animals exposed to *E*. *coli* relative to control animals grown on *E*. *coli*. **B.** qRT-PCR analysis of SKN-1-dependent genes in *rpt-6(RNAi)* or *elt-2(RNAi)* animals exposed to *P*. *aeruginosa* for 12 hours relative to control animals infected with *P*. *aeruginosa* for 12 hours. **C.** Control, *elt-2(RNAi)*, *rpt-6(RNAi)*, and *rpt-6*;*skn-1* co-RNAi animals were exposed to *P*. *aeruginosa* and scored for survival, ***P<0.0001, NS = not significant. All bars represent means ± SEM; n = 3 (t-test *P<0.05, **P<0.01 ***P<0.001).

We observed that although *rpt-6(RNAi)* animals were significantly susceptible to *P*. *aeruginosa* infection, they were not as susceptible to the pathogen as *elt-2(RNAi)* animals (Fig 1D). However, *rpt-6(RNAi)* animals exhibited a downregulation of immune genes comparable to that of *elt-2(RNAi)* animals (Fig 1C). SKN-1/Nrf is a transcription factor that is involved in the response to oxidative stress and protection against bacterial infection via the PMK-1/p38 MAPK pathway [24]. Because SKN-1 is known to be activated when the proteasome system is perturbed by the inactivation of proteasome genes [25,26], we reasoned that SKN-1 activation might confer some protection against *P*. *aeruginosa* infection in *rpt-6*(*RNAi*) animals. Therefore, we examined the expression of selected SKN-1 target genes [24]. We found that in the absence of infection, *gst-4*, *gst-5*, and *gst-10* were upregulated in *rpt-6(RNAi)* animals compared with the control animals (Fig 2A). Upon exposure to *P*. *aeruginosa*, the expression of *gst-4* and *gst-5* was still upregulated in *rpt-6*(*RNAi*) animals. In contrast, the expression of these SKN-1 reporter genes was significantly downregulated in *elt-2*(*RNAi*) animals during infection (Fig 2B). Because *skn-1* transcripts were not upregulated in *rpt-6(RNAi)* (Fig 2A; Fig 2B), SKN-1 might be activated post-transcriptionally by the disruption in the proteasome caused by *rpt-6* inhibition. To confirm the idea that SKN-1 partially protects *rpt-6*(*RNAi*) animals from *P*. *aeruginosa* infection, we used RNAi to inhibit both *rpt-6* and *skn-1*. As shown in Fig 2C, these animals were as susceptible to *P*. *aeruginosa* infection as *elt-2*(*RNAi*) animals, indicating that activation of SKN-1 partially compensated for the immune deficiency of *rpt-6(RNAi)* animals via an ELT-2-independent mechanism.

### Inhibition of proteasome activity does not attenuate ELT-2-mediated transcription during infection

**Fig 3:**
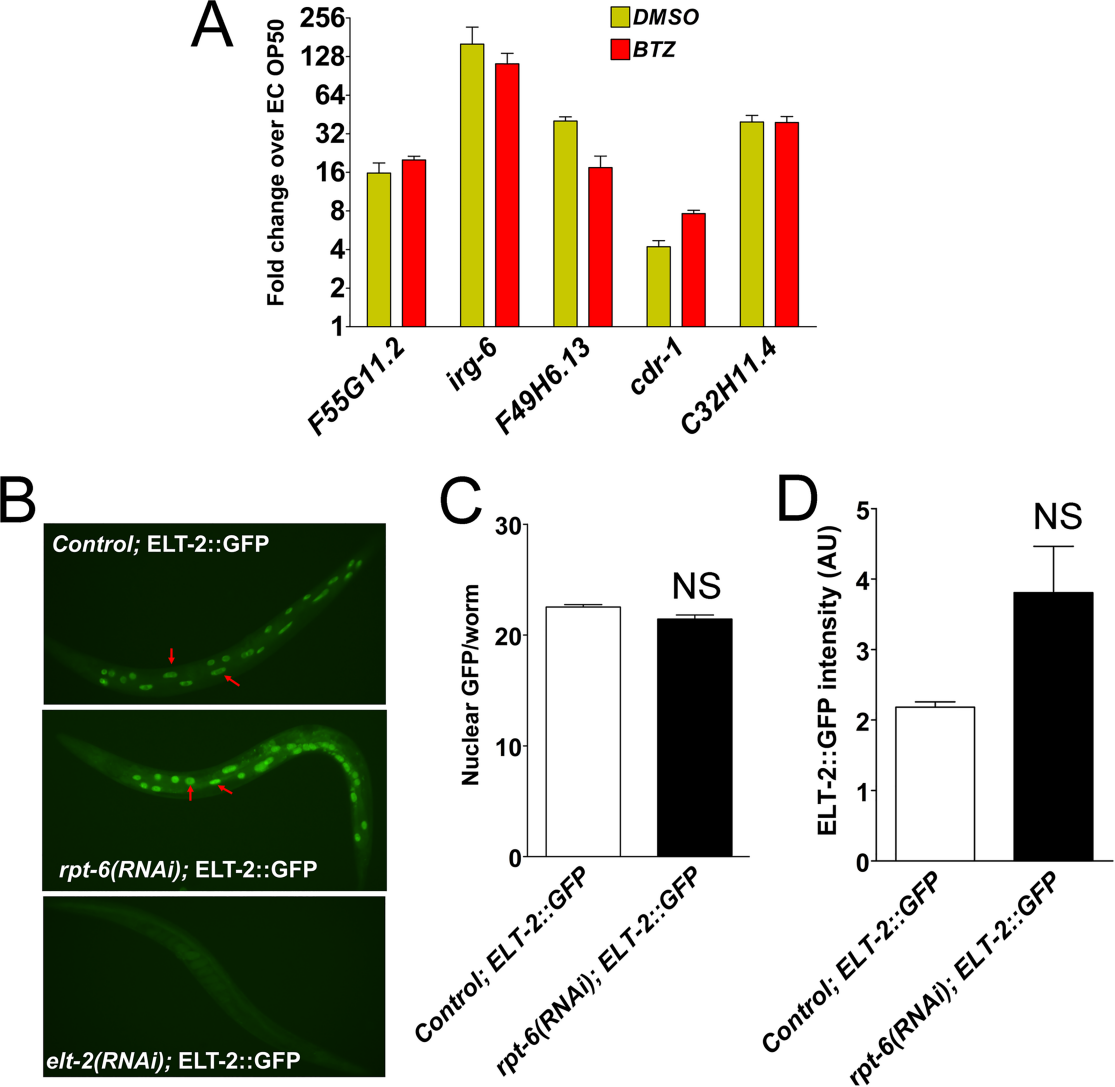
Immune activation of ELT-2-dependent genes is independent of proteasome activity. **A.** qRT-PCR analysis of immune genes in bortezomib (BTZ) and DMSO-treated animals exposed to *P*. *aeruginosa* for 4 hours relative to control animals exposed to *E*. *coli*. Error bars indicate means ± SEM; n = 3 (t-test *P<0.05, **P<0.01, ***P<0.001). **B.** Nuclear ELT-2::GFP in control or *rpt-6(RNAi)* animals. **C.** Quantification of number of nuclear ELT-2::GFP in control and *rpt-6(RNAi)* animals. **D.** Quantification of nuclear ELT-2::GFP intensity (per animal) in control and *rpt-6(RNAi)* animals. Fluorescence was quantified using ImageJ software. Shown are results of three biological replicates, all bars represent means ± SEM; n = 13 (t-test, NS = not significant).

Because ELT-2-dependent genes are downregulated during infection in *rpt-6*(*RNAi*) animals, we wanted to investigate whether this downregulation of ELT-2 target genes was linked to the inhibition of proteasomal activity. We inhibited the proteasome activity using the proteasome inhibitor bortezomib. First, we ascertained that the proteasome was indeed inhibited in *rpt-6*(*RNAi*) animals as well as in bortezomib-treated animals by using the *sur-5*::*UbV-gfp* reporter strain. Normally, inhibition of the ubiquitin–proteasome system (UPS) leads to stabilization of the UbV-GFP fusion protein due to the absence of protein degradation [27]. RNAi of *rpt-6* in the reporter strain led to an elevated UbV-GFP fluorescent signal (S6A and S6B Fig), indicating an inhibition of proteasome activity in *rpt-6(RNAi)* animals. Likewise, treatment of the reporter strain with bortezomib resulted in the stabilization of the UbV-GFP fluorescent signal (S6C–S6F Fig), indicating inhibition of the proteasomal activity. However, similar treatment with bortezomib had no significant effect on the expression of ELT-2-dependent immune genes activated during *P*. *aeruginosa* infection (Fig 3A). These results indicate that inhibition of proteasome activity is unlikely to impact ELT-2 transcriptional activation of immune genes during infection.

Next, we investigated whether *rpt-6* RNAi prevented ELT-2 transcriptional activity by altering its nuclear localization. We utilized an *elt-2*::*gfp* reporter strain to quantify both the numbers of nuclei as well as the intensity of GFP fluorescence. As expected, *elt-2*(*RNAi*) animals did not show ELT-2::GFP expression, but ELT-2::GFP nuclear localization was still present in *rpt-6*(*RNAi*) animals (Fig 3B). The estimation of nuclear ELT-2::GFP in *rpt-6*(*RNAi*) animals showed that both the numbers and intensity of nuclear ELT-2::GFP were not significantly different from those observed in control animals (Fig 3C; Fig 3D). Taken together, these results showed that the proteolytic function of the proteasome did not mediate the ELT-2 transcriptional activation of immune genes.

### RPT-6 physically interacts with ELT-2/GATA

**Fig 4:**
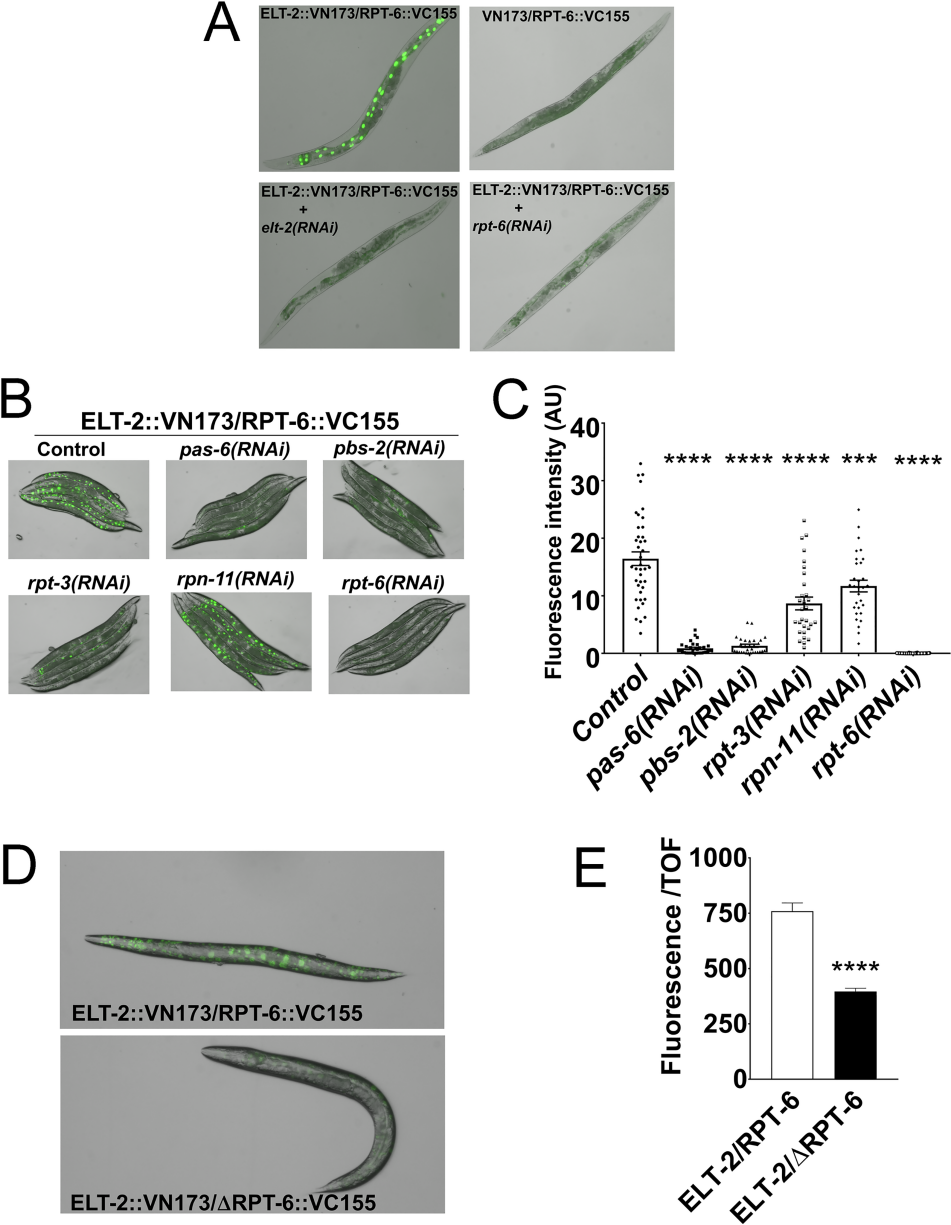
ELT-2 and RPT-6 physically interact *in vivo*. **A.** Bimolecular fluorescence complementation (BiFC) signals of animals co-expressing ELT-2::VN173 and RPT-6::VC155. Animals carrying RPT-6::VC155 and VN173 without ELT-2 were used as control. RNAi inhibition of *elt-2* and *rpt-6* in ELT-2::VN173/RPT-6::VC155 was also used as control. **B.** BiFC signals of transgenic animals co-expressing ELT-2::VN173 and RPT-6::VC155 or RNAi-treated animals. **C.** Quantification of bimolecular fluorescence signals of transgenic animals co-expressing ELT-2::VN173 and RPT-6::VC155 and RNAi-treated animals. The results represent three independent experiments containing a total of 150 animals (one-way ANOVA Dunnett's multiple comparisons test; **** P<0.0001, *** P = 0.0002). **D.** BiFC signals of transgenic animals co-expressing ELT-2::VN173 with RPT-6::VC155 or ΔRPT-6::VC155. **E.** Quantification of bimolecular fluorescence signals of transgenic animals co-expressing ELT-2::VN173 with RPT-6::VC155 or ΔRPT-6::VC155. Shown are three independent experiments combined, with a minimum of 399 animals used in each case. Error bars indicate means ± SEM (one-way ANOVA test; **** P<0.0001). Quantification was done using the Copas Biosort instrument (Union Biometrica, Holliston, MA).

Our co-immunoprecipitation and proteomic analysis indicated that ELT-2 and RPT-6 physically interacted to control gene expression. To confirm the ELT-2/RPT-6 interaction, we employed a bimolecular fluorescence complementation (BiFC) assay, which allows for the determination of physical interactions of proteins in living cells through direct visualization [28,29]. The BiFC constructs are engineered to individually express, in response to heat-shock, GFP protein fragments translationally fused with RPT-6 and ELT-2. Generally, interacting proteins bring the non-fluorescent fragments into close proximity for reconstitution and fluorescence. Twelve hours after heat shock, we observed fluorescence, indicating a physical interaction between RPT-6 and ELT-2 *in vivo* (Fig 4A). Animals carrying BiFC constructs without *elt-2* did not exhibit fluorescence. Knockdown of either *rpt-6* or *elt-2* by RNAi resulted in the absence of fluorescence, further confirming that the presence of the two proteins is required for the GFP reconstitution (Fig 4A). We wondered whether the ability of other components of the proteasome complex to affect ELT-2 activity may be a consequence of these components affecting the ELT-2/RPT-6 interaction. RNAi of selected proteasome genes reduced the extent of the ELT-2/RPT-6 *in vivo* interaction to varying degrees (Fig 4B; Fig 4C).

The ATPase activity of the 19S proteasome has been implicated in the mediation of the interaction between proteasome subunits and transcription factors [30,31]. Thus, we asked whether the active site of the ATPase domain of RPT-6 played a role in mediating the interaction with ELT-2 *in vivo*, and we found that GFP fluorescence is significantly reduced when a single amino acid in the ATPase domain of RPT-6 is mutated (Fig 4D; Fig 4E). Taken together, these results indicate that the ATPase activity of RPT-6 is needed for its interaction with ELT-2 *in vivo* and that this interaction required other components of the 26S proteasome complex.

## Discussion

ELT-2/GATA transcription plays a crucial role in the control of immunity in *C*. *elegans* against both bacterial and fungal pathogens by cooperating with the PMK-1/p38 MAPK and SKN-1/Nrf pathways [3,5,32]. However, no information is available concerning the proteins that interact with ELT-2 to participate in the control of gene expression. It is known that transcription factors can recruit and associate with co-regulatory proteins during gene transcription. In mammalian cells, the immunoproteasome, the proteasome-isoform of the standard proteasome, is known to mediate the immune response, but mainly through its proteolytic function, due to its higher degradation capability [33]. Here we showed that RPT-6, a component of 19S, could non-proteolytically mediate ELT-2 transcriptional activity and physically interact with ELT-2.

Our findings showed that inactivation of *rpt-6* inhibited the expression of ELT-2 target genes during infection. Our previous work has demonstrated that ELT-2 activates not only immune genes in response to pathogens, but also genes responsible for recovery from acute bacterial infections. Our new findings indicate that mediation of ELT-2 transcriptional activity by the proteasome is not limited to the immune response, but it is also involved in the recovery from bacterial infection. Inhibition of proteasome activity by bortezomib did not prevent the transcriptional activation of ELT-2 target genes during infection, indicating that RPT-6 control of ELT-2 targets is unlikely related to the proteolytic activity. We cannot completely rule out the possibility that a repressor of ELT-2, that is commonly targeted for proteolysis, reduces ELT-2 activity in *rpt-6(RNAi)* animals. However, we believe that the failure of bortezomib to alter ELT-2-mediated gene expression at the same concentrations at which it significantly inhibits the proteasome, makes this possibility unlikely.

Accumulating evidence indicates that different components of the proteasome interact with transcription factors to control gene expression in a manner that is independent of the canonical proteasomal activity [8,16,17,34,35]. The current notion is that components of the proteasome are recruited to the site of transcription for the subsequent mobilization of other transcriptional machinery, including chromatin remodeler and stabilization of enhanceosome at the site of transcription [16,17,34,35]. Similarly, our findings show that the inactivation of RTP-6 affects ELT-2-mediated transcription. Our results also suggest that other components of the proteasome affect both ELT-2 transcriptional activity and the ELT-2/RPT-6 interaction *in vivo*. Indeed, the three subunits of the proteasome have been shown to interact in yeast with activated GAL10, a galactose metabolism structural component [34].

Perturbation of core cellular activities, including proteasomal function, induces the expression of detoxification and innate immune response genes [36]. Here we show that inhibition of the RPT-6 subunit of the proteasome represses the expression of innate immune genes. Our results indicate that this effect is not related to the proteasomal activity, but rather due to the lack of physical interaction between RPT-6 and ELT-2. Thus, disruptions in the proteasome have different effects on other transcription factors. Proteasomal perturbations upregulate the expression of genes that are reporter of the activity of other transcription factors, including EGL-9, ZIP-2, ELT-3 and SKN-1 [26,36–39]. Consistently with these observations, we found that SKN-1-controlled genes were induced in *rpt-6(RNAi)* animals.

In conclusion, our results demonstrate that the proteasome is an important player in transcriptional activation during the immune response in *C*. *elegans*. We identified RPT-6, a subunit of the 19S proteasome, as the binding partner of the ELT-2/GATA transcription factor. Inactivation of *rpt-6* and other components of the proteasome attenuates the activation of immune genes that are regulated by ELT-2. However, proteasome inhibition does not abolish the activation of immune genes, indicating that this control of innate immunity by the proteasome occurs in a non-proteolytic manner. In addition, ELT-2 and RPT-6 physically interact, a phenomenon that is affected by other components of the proteasome and that it requires the ATPase activity of RPT-6. It is noteworthy that both ELT-2/GATA and the proteasome are evolutionarily conserved. The two closest homologs to ELT-2 in humans, GATA6 and GATA4, also participate in the control of immunity. The former possesses an immune protective function against *P*. *aeruginosa* infection in human lung epithelial cells, while the latter affects intestinal immunity [4,40]. The non-proteolytic interaction between the proteasome and ELT-2 described herein may be part of a conserved mechanism involved in the control of immune responses.

## Materials and methods

### *C*. *elegans* and bacterial strains

All *C*. *elegans* strains used were grown and maintained on standard NGM-OP50 plates at 15°C. Bristol N2, HH142 [*fer-1(b232ts)*], OP56 gaEx290 [*elt-2*::TY1::EGFP::3xFLAG(92C12) + unc-119(+)], SD1949 *glo-4*(ok623) V; gaIs290 [*elt-2*::TY1::EGFP::3xFLAG(92C12) + unc-119(+)] were obtained from the Caenorhabditis Genetics Center. *P*_*F55G11*.*2*_::*gfp* worms were provided by Michael Shapira (University of California Berkeley, Berkeley, California), and hhIs64 *[unc-119(+); sur-5*::*UbV-GFP]III; hhIs73 [unc-119(+); sur-5*::*mCherry]* was constructed by T. Hoppe, University of Cologne. The following bacterial strains were used in this study: *E*. *coli* OP50-1 [Sm^R^], *P*. *aeruginosa* PA14, and E. *coli* strain HT115 pL4440-RNAi (HT115-RNAi) [Amp^R^, Tet^R^]. In all experiments, *fer-1* animals were grown at the 15°C permissive temperature to gravid adults. Egg laying and subsequent growth for assays were carried out at the 25°C non-permissive temperature.

### Co-immunoprecipitation and proteomics

Strain OP56 carrying the ELT-2::GFP transgene was crossed with *fer-1* to obtain the transgenic strain with *fer-1* background, this was done to avoid progeny issues in the subsequent assay. Approximately 2,000 young adult animals grown at 25°C were washed with M9 and transferred unto the *P*. *aeruginosa* PA14 plate for 12 hours at 25°C. The animals were harvested, washed several times in M9 buffer, and frozen in PBS buffer. The worm pellet was later sonicated, and the proteins were immunoprecipitated using GFP-Trap A beads (Chromotek, Germany) at 4°C for 4 hours following the manufacturer’s guidelines. The complexes were washed with 50 mM NH_4_HCO_3_ and sent to Duke University Proteomics Core Facility for proteomic analysis. In-gel trypsin digestion was performed according to a standard protocol (https://genome.duke.edu/sites/genome.duke.edu/files/In-gelDigestionProtocolrevised_0.pdf). Qualitative LC/MS/MS was performed on the sample using a nanoAcquity UPLC system (Waters Corp) coupled to a Thermo QExactive Plus high-resolution accurate mass tandem mass spectrometer (Thermo). Raw data were processed using the Mascot Distiller and Mascot Server (v2.5, Matrix Sciences), and Scaffold v4 (Proteome Software, Inc) was used for curation. The protein and peptide threshold FDR were set to 5% and 1%, respectively.

### Gene knockdown by RNA interference

Bacterial clones obtained from the Ahringer library and empty RNAi vector (EV) were grown in LB broth containing 100 μg/ml ampicillin at 37°C for 9 hours, concentrated, and spread onto NGM plates containing 100 μg/ml ampicillin plus 3 mM isopropyl 1-thio-β-D-galactopyranoside. RNAi-expressing bacteria were incubated at 37°C overnight and further overnight at room temperature to produce a thick bacterial lawn. Because most of the genes were lethal when knocked down during the larval stage, RNAi was initiated at L4, excluding skn-1, which was started during egg laying. Young adult *fer-1* animals grown at the 25°C non-permissive temperature for 36 hours were fed bacteria expressing dsRNA for 36 hours at 25°C. Co-RNAi was performed by mixing the respective bacteria clones 1:1 before seeding. The nematodes were then used for subsequent assays. Control animals were grown on empty vector in all cases. All RNAi clones were verified by DNA sequencing.

### *P*. *aeruginosa* killing and recovery assays

*P*. *aeruginosa killing*: PA14 grown in LB broth for 15 hours was seeded onto modified NGM agar medium (0.35% instead of 0.25% peptone), and the plates were incubated overnight at 37°C. Synchronized animals were transferred onto PA14-seeded plates and incubated at 25°C. Animals were transferred onto a new pathogen lawn every day and scored every 12 hours. Animals were considered dead when they failed to respond to touch. Each experiment performed independently in triplicate, consisting of 50 animals in each experiment.

*P*. *aeruginosa recovery assay*: After infection for 12 hours as described above, infected or control animals grown on *E*. *coli* OP50 were rinsed by transferring them unto 120 μl M9 plus 300 μg/ml streptomycin on NGM-*E*. *coli* OP50 plates. The animals were allowed to swim out of the solution and unto the OP50 lawn, and they were subsequently transferred to modified NGM plates containing 300 μg/ml streptomycin seeded with *E*. *coli* OP50. Animals were transferred onto new plates containing antibiotics and seeded with *E*. *coli* OP50 daily and scored every 24 hours. Animals were considered dead when they failed to respond to touch. All experiments were performed independently in triplicate, consisting of 50 animals in each experiment.

### RNA isolation and qRT-PCR

Approximately 2,000 RNAi or control animals were washed with M9, transferred onto *P*. *aeruginosa* PA14-modified NGM plates for 12 hours at 25°C, and harvested in M9 buffer. For recovery, after12 hours of infection, animals were washed with several changes of M9 and M9 plus 300 μg/ml streptomycin and transferred onto *E*. *coli* OP50 plates containing 300 μg/ml streptomycin for 6 hours at 25°C. Harvested animals were washed with M9 and frozen in TRIzol (Life Technologies, Carlsbad, CA). Total RNA extraction and qRT-PCR using 2 μg total RNA were carried out as previously described [41]. All primer sequences used are available upon request. Primer sequences used for SKN-1 targets were from Hoeven *et al*. [24].

### Proteasomal inhibition and infection

Bortezomib (Millipore, Temecula, CA, USA), a proteasome inhibitor, dissolved in DMSO to a stock concentration of 5 mM (w/v), was spread onto three-day-old NGM *E*. *coli* OP50-seeded plates to a final concentration of 100 nM. Control plates contained only DMSO or NGM *E*. *coli* OP50. All plates contained a final concentration of 0.002% DMSO. Approximately 1,500 L4 stage animals were added onto individual plates and incubated for 13 hours at 20°C. Because bortezomib is a reversible inhibitor, to ensure that the proteasome was still inhibited, we flooded the overnight incubated PA14-seeded plates with bortezomib (100 nM final concentration) or DMSO. Drug and DMSO-treated animals were transferred onto PA14-seeded plates, while the animals grown on NGM *E*. *coli* OP50 only were transferred onto *E*. *coli* OP50 plates. All plates were incubated at 25°C for 4 hours. After infection, animals were harvested, rinsed, and frozen in TRIzol (Life Technologies, Carlsbad, CA) for RNA isolation.

### Bimolecular fluorescence complementation (BiFC) and plasmid construction

To construct plasmids for the BiFC assay for protein interaction, *elt-2* and *rpt-6* cDNA (GE Healthcare Dharmacon Inc.) were subcloned into pCE-BiFC-VN173 and pCE-BiFC-VC155 plasmids (Addgene, Cambridge, MA), respectively, both of which contain the heat shock promoter P*hsp-16*.*41*. Full-length *elt-2* cDNA was subcloned in-frame into pCE-BiFC-VN173 between SmaI and AgeI, while the full-length *rpt-6* cDNA was also subcloned in-frame into pCE-BiFC-VC155 between SmaI and KpnI. The generation of the RPT-6 ATPase mutant construct was performed using site-directed mutagenesis on the pCE-BiFC-*rpt-6*::VC155 to replace lysine at position 206 of RPT-6 with arginine. The BiFC plasmid constructs were injected into N2 worms at 15 ng/μl each, together with pRF4(rol-6) at 100 ng/μl (co-injection marker) [29]. To detect the interaction, transgenic worms carrying the BiFC plasmid constructs were raised to young adults at 20°C, heat shocked for 3 h at 33°C, and allowed to recover for 12 hours at 20°C. Direct visualization of fluorescent signals of the induced expression of fusion proteins (ELT-2 and RPT-6) was captured using a Leica M165 FC fluorescence stereomicroscope. The BiFC assay involving RNAi of other components of the 26S for estimation of the ELT-2/RPT-6 interaction was performed independently in triplicate with 50 animals. One-way ANOVA Dunnett's multiple comparisons test was employed for evaluation.

## Supporting information

S1 Fig *P*. *aeruginosa* infection and recovery assays on RNAi of candidate genes for which proteins were co-immunoprecipitated with ELT-2 during infection. **A.** Control, *elt-2(RNAi)*, or animals where candidate interacting genes (*rpt-6*, *W10C8*.*5*, *rab-8*, *eif-3*.*E*, *tba-4*, *gpb-1*, *unc-52*, *F48E8*.*3*, *hsp-12*.*2*, and *K02D7*.*1*) have been knocked down by RNAi were exposed to *P*. *aeruginosa* and scored for survival.
**B.** Control, *elt-2(RNAi)*, or animals where candidate interacting genes (*rpt-6*, *W10C8*.*5*, *rab-8*, *eif-3*.*E*, *tba-4*, *gpb-1*, *unc-52*, *F48E8*.*3*, *hsp-12*.*2*, and *K02D7*.*1*) have been knocked down by RNAi were exposed to *P*. *aeruginosa* for 12 hours, treated with streptomycin, and then transferred to *E*. *coli* plus Streptomycin plates and scored for survival. Scoring started 24 hours post initial exposure to *P*. *aeruginosa*.
(TIF)

S2 Fig *elt-2* and *rpt-6* co-regulate the expression of innate immune genes. qRT-PCR analysis of immune genes in *elt-2(RNAi)* or *elt-2(RNAi); rpt-6(RNAi)* animals exposed to *P*. *aeruginosa* for 12 hours relative to control animals exposed to *P*. *aeruginosa*. Error bars indicate means ± SEM; n = 3 (NS = not significant).
(TIF)

S3 Fig *elt-2* and *rpt-6* knockdown in single- or co-RNAi treated animals. **A.** Nuclear expression of ELT-2::GFP in control, *elt-2(RNAi)*, or *elt-2(RNAi); rpt-6(RNAi)* animals.
**B.** qRT-PCR quantification of *rpt-6* in *rpt-6(RNAi) or elt-2(RNAi)*; *rpt-6(RNAi)* animals exposed to *P*. *aeruginosa* for 12 hours relative to control animals exposed to *P*. *aeruginosa*. Error bars indicate means ± SEM; n = 3.
(TIF)

S4 Fig *elt-2* and *rpt-6* control lifespan. Control, *elt-2(RNAi)*, or *rpt-6(RNAi)* animals were placed on NGM plates of heat-killed *E*. *coli* OP50 supplemented with antibiotics (100 μg/ml streptomycin, 50μg/ml kanamycin and 10 μg/ml Nystatin) and scored for survival at 20°C (N = 240 per group, ***P<0.0001).
(TIF)

S5 Fig Different components of the 26S proteasome transcriptionally affect ELT-2. **A.** Fluorescence images of control, *elt-2(RNAi)*, *rpt-3(RNAi)*, *pbs-2(RNAi)*, *pas-6(RNAi)*, and *rpn-11(RNAi)* animals expressing *P*_*F55G11*.*2*_::*gfp*. Control or RNAi treated *P*_*F55G11*.*2*_::*gfp* animals were transferred to *E*. *coli* OP50 and later visualized using a Leica M165 FC fluorescence stereomicroscope.
**B.** qRT-PCR analysis of immune genes in control, *elt-2(RNAi)*, or *rpn-11(RNAi)* animals exposed to *P*. *aeruginosa* for 12 hours relative to control animals exposed to *E*. *coli*. Red bars correspond to gene expression in control animals exposed to *P*. *aeruginosa* for 12 hours relative to control animals exposed to *E*. *coli*. Error bars indicate means ± SEM; n = 3 (t-test **P<0.01, ***P<0.001).
**C.** Control, *elt-2(RNAi)*, and *rpn-11(RNAi)* animals were exposed to *P*. *aeruginosa* and scored for survival, ***P<0.0001.
(TIF)

S6 Fig Proteasomal inhibition led to the stabilization of ubiquitin-conjugated protein. **A.** Fluorescence images of control and *rpt-6(RNAi)* animals expressing UbV::GFP fusion protein and mCherry protein under the control of the *sur-5* promoter.
**B.** Fluorescence quantification of UbV::GFP in control and *rpt-6(RNAi)* animals. Error bars indicate means ± SEM; n = 3 (t-test **P<0.01). Quantification was done using ImageJ software.
**C.** Fluorescence images of DMSO (control) and bortezomib (BTZ)-treated animals expressing UbV::GFP fusion protein and mCherry protein under the control of the *sur-5* promoter.
**D.** Fluorescence quantification of UbV::GFP in DMSO (control) and bortezomib (BTZ)-treated animals. Error bars indicate means ± SEM (one-way ANOVA test, ***P<0.0001). Each group contained 125 animals. Quantification was done using ImageJ software.
**E-F.** Fluorescence quantification of UbV::GFP in DMSO (control) and bortezomib (BTZ)-treated animals, normalized to both mcherry and adult animal size (time of flight; TOF). Error bars indicate means ± SEM (one-way ANOVA test, ***P<0.0001). Each group contained 548 animals. Quantification was done using the Copas Biosort instrument (Union Biometrica, Holliston, MA).
(TIF)

S1 Table List of candidate pull-down proteins interacting with ELT-2 during *P*. *aeruginosa* infection. The human homolog for these proteins was retrieved from wormbase using the simplemine tool. N.A; not available.
(DOCX)
