# Supplementary figures and images for: Non-proteolytic activity of 19S proteasome subunit RPT-6 regulates GATA transcription during response to infection
Source: PLoS Genet. 2018 Sep 28;14(9):e1007693. doi: 10.1371/journal.pgen.1007693 (PMC6179307; doi:10.1371/journal.pgen.1007693)

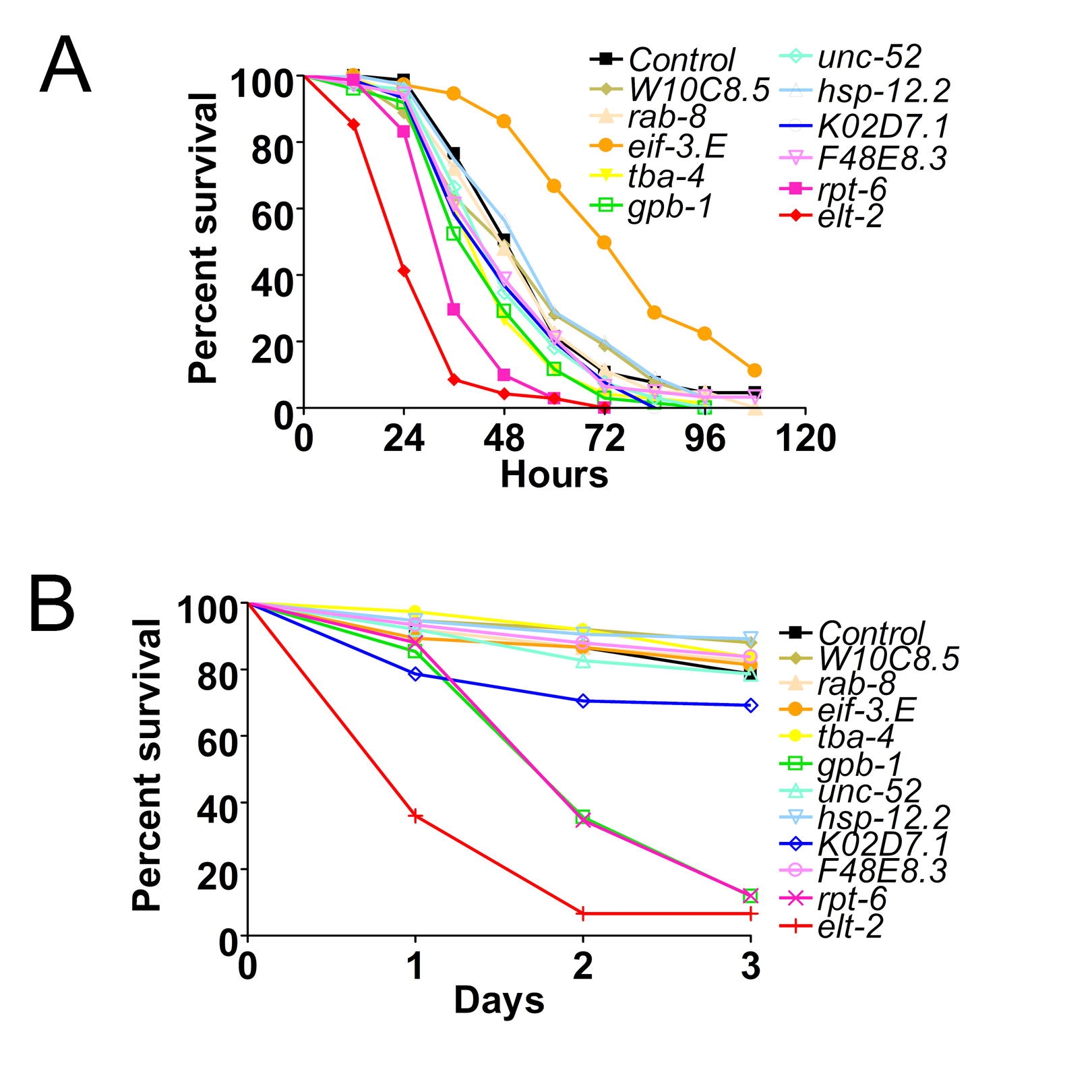

Supplement: S1 Fig — A. Control, elt-2(RNAi), or animals where candidate interacting genes (rpt-6, W10C8.5, rab-8, eif-3.E, tba-4, gpb-1, unc-52, F48E8.3, hsp-12.2, and K02D7.1) have been knocked down by RNAi were exposed to P. aeruginosa and scored for survival. B. Control, elt-2(RNAi), or animals where candidate interacting genes (rpt-6, W10C8.5, rab-8, eif-3.E, tba-4, gpb-1, unc-52, F48E8.3, hsp-12.2, and K02D7.1) have been knocked down by RNAi were exposed to P. aeruginosa for 12 hours, treated with streptomycin, and then transferred to E. coli plus Streptomycin plates and scored for survival. Scoring started 24 hours post initial exposure to P. aeruginosa. (TIF) [file pgen.1007693.s001.tif]

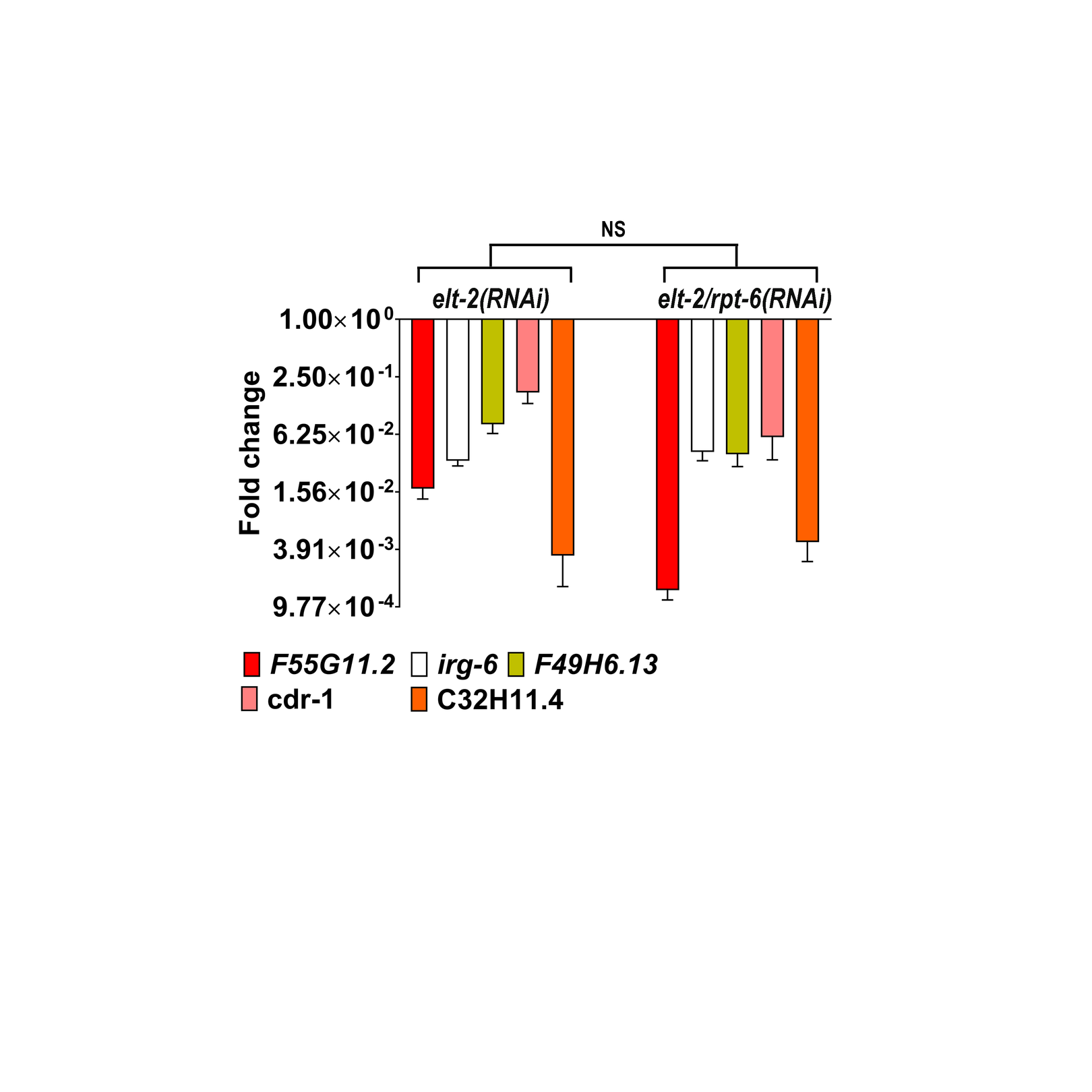

Supplement: S2 Fig — qRT-PCR analysis of immune genes in elt-2(RNAi) or elt-2(RNAi); rpt-6(RNAi) animals exposed to P. aeruginosa for 12 hours relative to control animals exposed to P. aeruginosa. Error bars indicate means ± SEM; n = 3 (NS = not significant). (TIF) [file pgen.1007693.s002.tif]

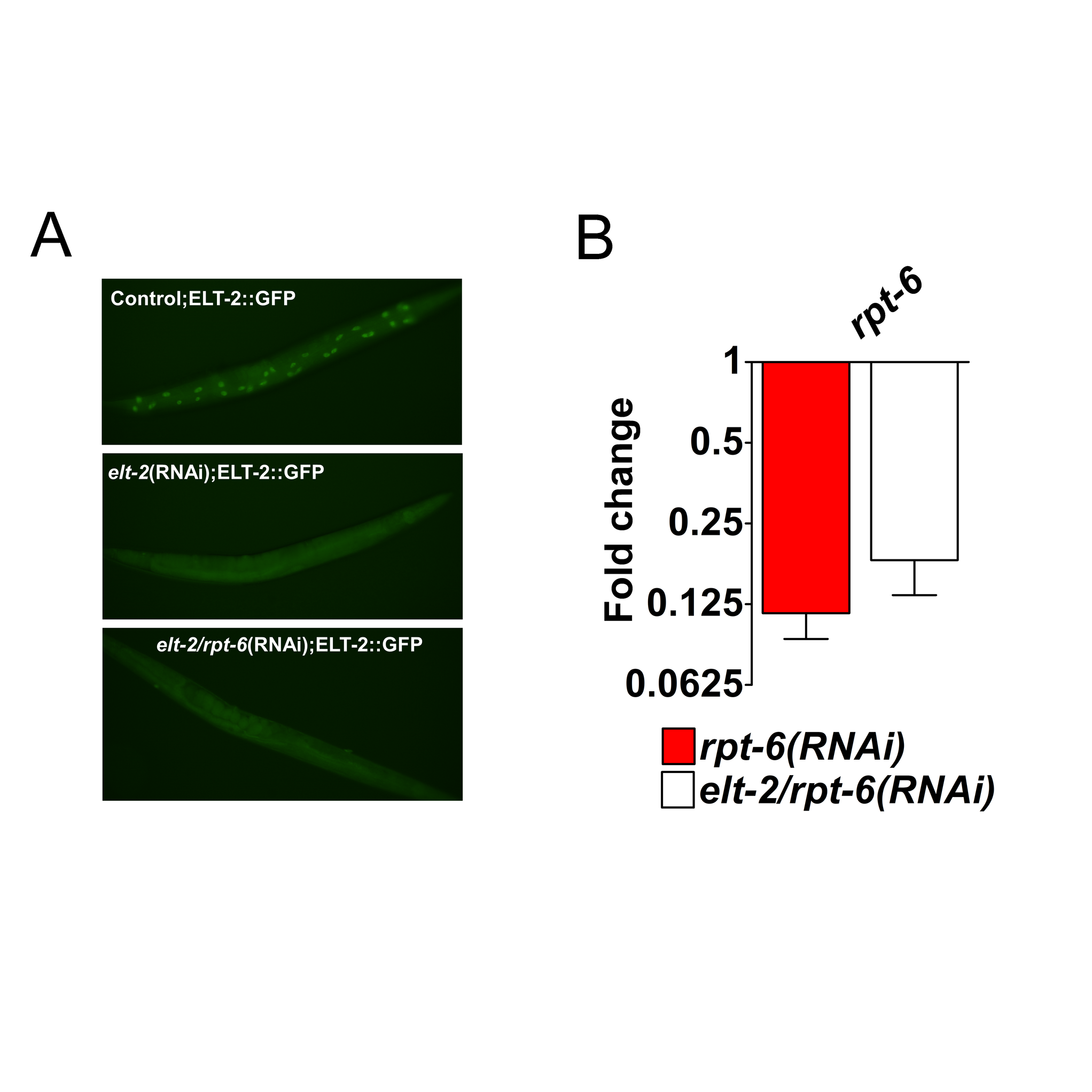

Supplement: S3 Fig — A. Nuclear expression of ELT-2::GFP in control, elt-2(RNAi), or elt-2(RNAi); rpt-6(RNAi) animals. B. qRT-PCR quantification of rpt-6 in rpt-6(RNAi) or elt-2(RNAi); rpt-6(RNAi) animals exposed to P. aeruginosa for 12 hours relative to control animals exposed to P. aeruginosa. Error bars indicate means ± SEM; n = 3. (TIF) [file pgen.1007693.s003.tif]

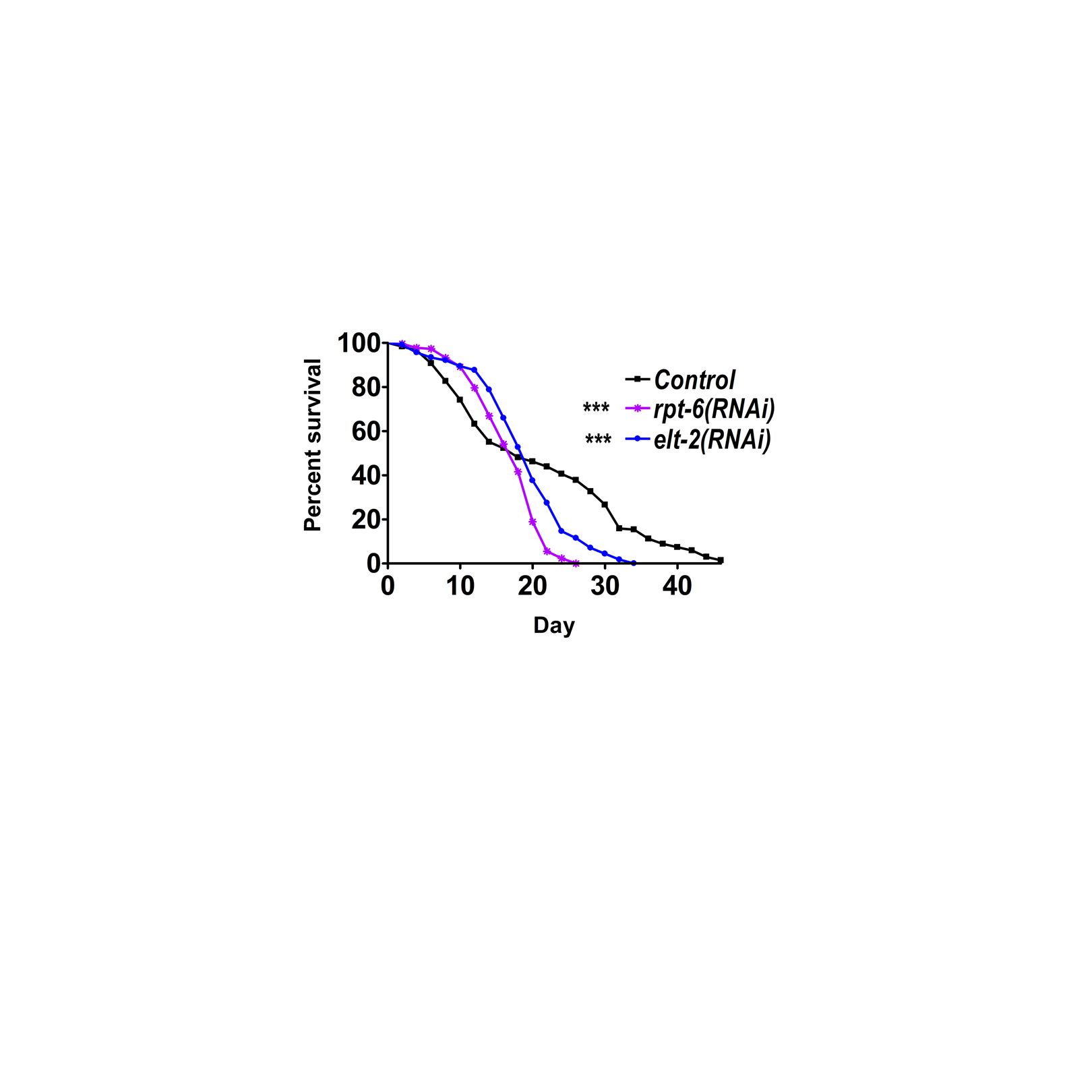

Supplement: S4 Fig — Control, elt-2(RNAi), or rpt-6(RNAi) animals were placed on NGM plates of heat-killed E. coli OP50 supplemented with antibiotics (100 μg/ml streptomycin, 50μg/ml kanamycin and 10 μg/ml Nystatin) and scored for survival at 20°C (N = 240 per group, ***P<0.0001). (TIF) [file pgen.1007693.s004.tif]

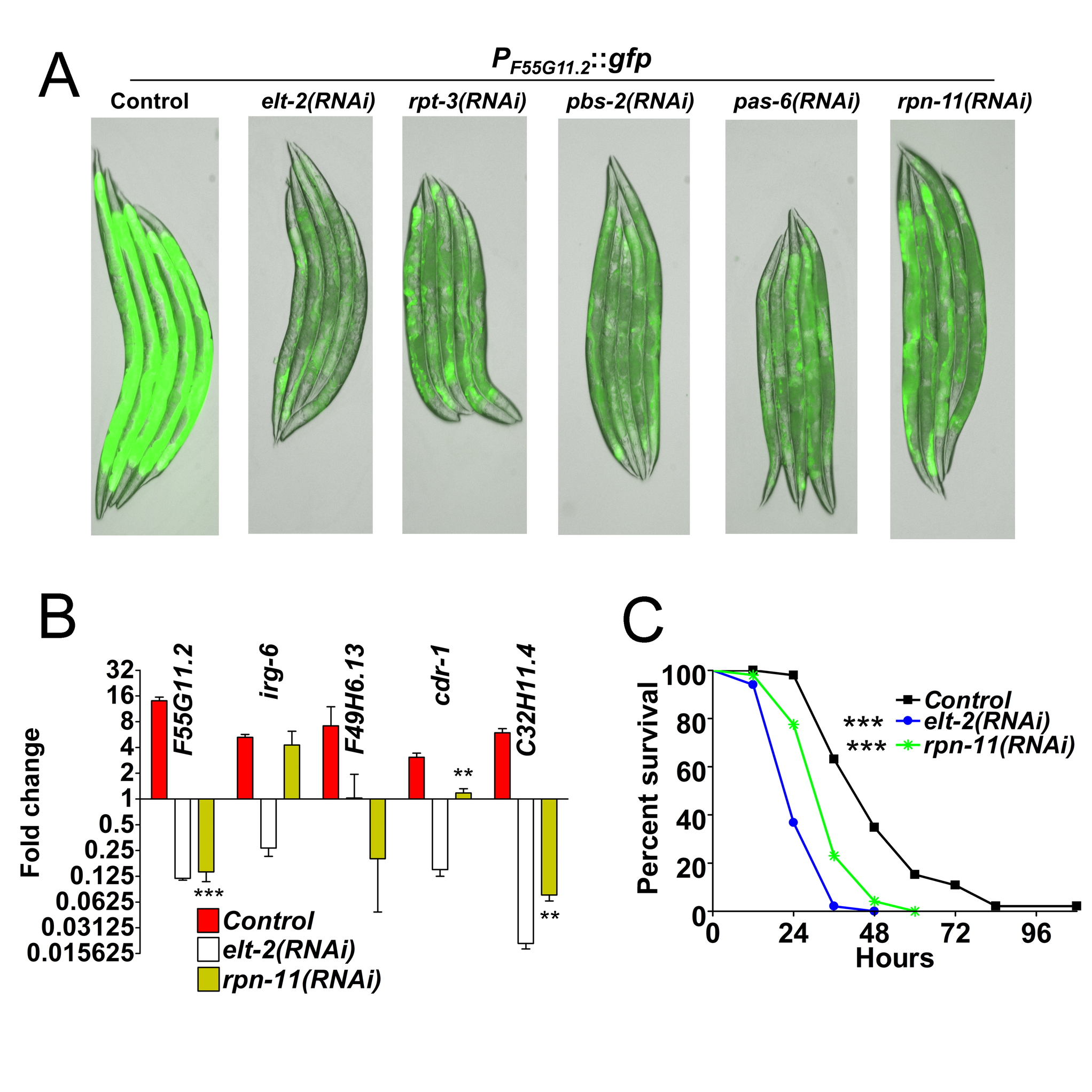

Supplement: S5 Fig — A. Fluorescence images of control, elt-2(RNAi), rpt-3(RNAi), pbs-2(RNAi), pas-6(RNAi), and rpn-11(RNAi) animals expressing PF55G11.2::gfp. Control or RNAi treated PF55G11.2::gfp animals were transferred to E. coli OP50 and later visualized using a Leica M165 FC fluorescence stereomicroscope. B. qRT-PCR analysis of immune genes in control, elt-2(RNAi), or rpn-11(RNAi) animals exposed to P. aeruginosa for 12 hours relative to control animals exposed to E. coli. Red bars correspond to gene expression in control animals exposed to P. aeruginosa for 12 hours relative to control animals exposed to E. coli. Error bars indicate means ± SEM; n = 3 (t-test **P<0.01, ***P<0.001). C. Control, elt-2(RNAi), and rpn-11(RNAi) animals were exposed to P. aeruginosa and scored for survival, ***P<0.0001. (TIF) [file pgen.1007693.s005.tif]

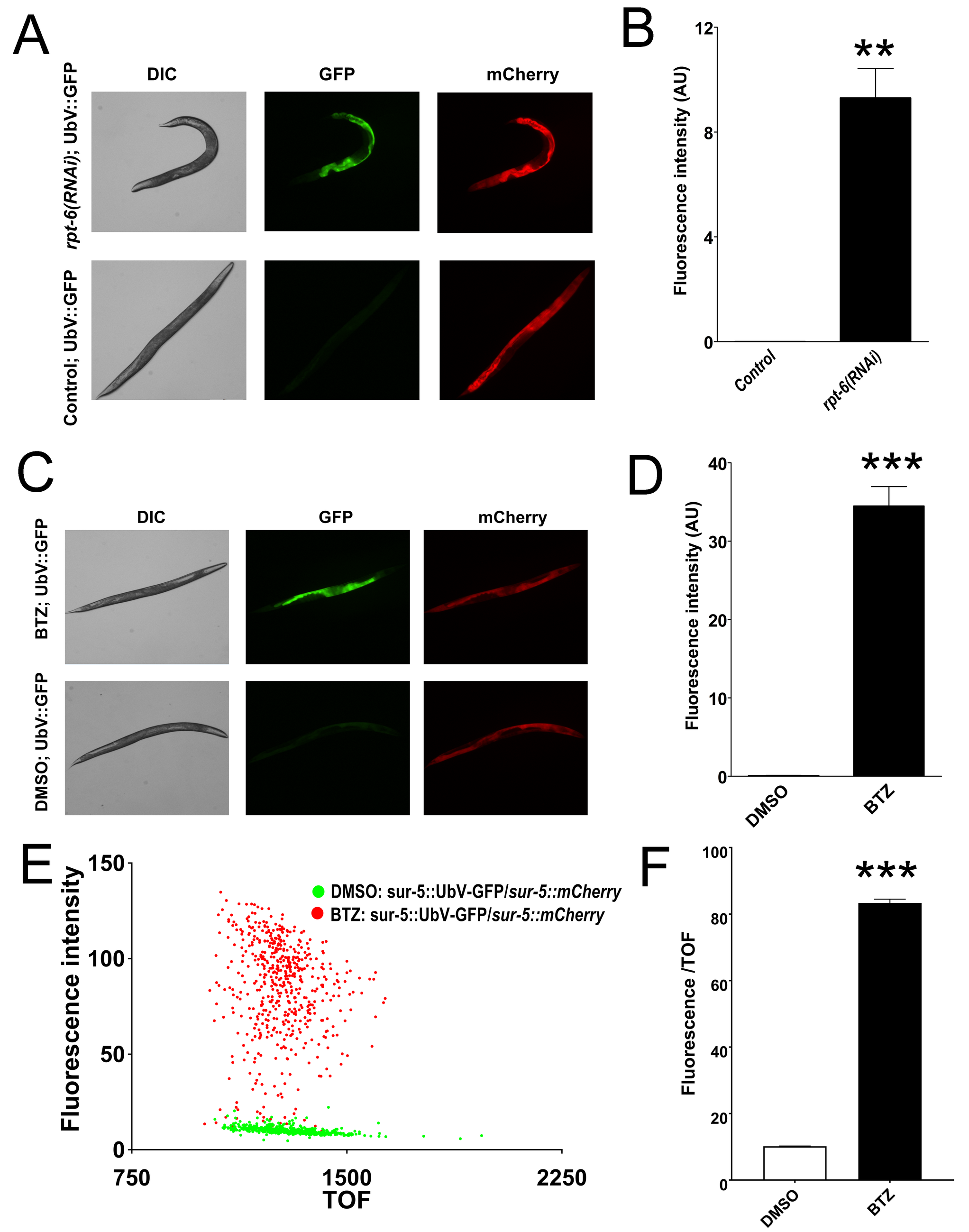

Supplement: S6 Fig — A. Fluorescence images of control and rpt-6(RNAi) animals expressing UbV::GFP fusion protein and mCherry protein under the control of the sur-5 promoter. B. Fluorescence quantification of UbV::GFP in control and rpt-6(RNAi) animals. Error bars indicate means ± SEM; n = 3 (t-test **P<0.01). Quantification was done using ImageJ software. C. Fluorescence images of DMSO (control) and bortezomib (BTZ)-treated animals expressing UbV::GFP fusion protein and mCherry protein under the control of the sur-5 promoter. D. Fluorescence quantification of UbV::GFP in DMSO (control) and bortezomib (BTZ)-treated animals. Error bars indicate means ± SEM (one-way ANOVA test, ***P<0.0001). Each group contained 125 animals. Quantification was done using ImageJ software. E-F. Fluorescence quantification of UbV::GFP in DMSO (control) and bortezomib (BTZ)-treated animals, normalized to both mcherry and adult animal size (time of flight; TOF). Error bars indicate means ± SEM (one-way ANOVA test, ***P<0.0001). Each group contained 548 animals. Quantification was done using the Copas Biosort instrument (Union Biometrica, Holliston, MA). (TIF) [file pgen.1007693.s006.tif]
