# Supplementary material for: Non-proteolytic activity of 19S proteasome subunit RPT-6 regulates GATA transcription during response to infection
Source: PLoS Genet. 2018 Sep 28;14(9):e1007693. doi: 10.1371/journal.pgen.1007693 (PMC6179307; doi:10.1371/journal.pgen.1007693)
Supplement: S1 Table — The human homolog for these proteins was retrieved from wormbase using the simplemine tool. N.A; not available. (DOCX) [file pgen.1007693.s007.docx]

| Public Name | Sequence Name | Human Ortholog | Annotation |
| --- | --- | --- | --- |
| W10C8.5 | W10C8.5 | ENSG00000223572 | Uncharacterized protein |
| RAB-8 | D1037.4 | ENSG00000166128 | RAB family |
| EIF-3.E | B0511.10 | ENSG00000104408 | Eukaryotic translation initiation factor 3 subunit E |
| SGT-1 | R05F9.10 | ENSG00000104969 | Small Glutamine-rich Tetratrico repeat protein |
| TBA-4 | F44F4.11 | ENSG00000167553 | Tubulin alpha chain |
| GPB-1 | F13D12.7 | ENSG00000114450 | Guanine nucleotide-binding protein subunit beta-1 |
| UNC-52 | ZC101.2 | ENSG00000142798 | Uncharacterized protein |
| F48E8.3 | F48E8.3 | N.A. | Uncharacterized protein |
| HSP-12.2 | C14B9.1 | ENSG00000109846 | Heat shock protein Hsp-12.2 |
| RPT-6 | Y49E10.1 | ENSG00000087191 | Proteasome Regulatory Particle, ATPase-like |
| K02D7.1 | K02D7.1 | ENSG00000198805 | Purine nucleoside phosphorylase |
| IARS-1 | R11A8.6 | ENSG00000196305 | Isoleucine--tRNA ligase, cytoplasmic |
| ACDH-7 | T25G12.5 | ENSG00000117054 | Acyl CoA DeHydrogenase |
| F55G11.4 | F55G11.4 | ENSG00000143819 | Uncharacterized protein |
